# The Optimization Study of Karst-Filling Clay-Cement Grout Based on Orthogonal Experiment and Regression Analysis

**DOI:** 10.3390/ma18091943

**Published:** 2025-04-24

**Authors:** Wenqin Yan, Chao Deng, Yuehui Cai, Aikun Chu, Shifan Shen, Xuanyu Wu

**Affiliations:** 1 Key Laboratory of Metallogenic Prediction of Nonferrous Metals and Geological Environment Monitoring, Ministry of Education, Changsha 410083, China; 235001037@csu.edu.cn (W.Y.); 225011106@csu.edu.cn (Y.C.); 225012142@csu.edu.cn (A.C.); 235011107@csu.edu.cn (S.S.); 235012138@csu.edu.cn (X.W.); 2 School of Geosciences and Info-Physics, Central South University, Changsha 410083, China; 3 College of Civil Engineering, Hunan City University, Yiyang 413000, China

**Keywords:** shield tunneling, karst grouting, mix optimization, orthogonal experiment, regression analysis

## Abstract

During shield tunnel construction, karst development along the tunnel axis and in the surrounding area frequently poses a significant threat to engineering safety. To address this challenge, this study proposes multiple grouting systems and systematically analyzes the key mechanical properties of five grout formulations through orthogonal experiments, identifying the optimal formulations for engineering applications. A predictive model was established using linear regression, and its accuracy was validated through independent single-factor experiments. The results indicate the following: (1) Water content is the primary factor influencing fluidity, with its significance varying by system composition. The lake mud-cement grout exhibits the highest compressive pstrength. Moderate sand addition enhances strength, but excessive amounts significantly reduce fluidity. Additives demonstrate system dependency: HY-4 effectively improves fluidity, while sodium silicate significantly increases strength but reduces fluidity. (2) The developed model demonstrates good goodness of fit, with coefficients of determination (R^2^) ranging from 0.74 to 0.95, without significant autocorrelation or multicollinearity. Validation experiments confirm the model’s high predictive accuracy, with overall trends consistent with the measured data. (3) The lake mud-cement grout (A3B1C3) is recommended for reinforcement projects prioritizing stability, achieving a 28-day compressive strength of 4.74 MPa. The on-site wet clay-cement grout (A2B3C1) is suitable for high-permeability formations, with a strength of 1.1 MPa and a fluidity of 292.5 mm, both exceeding standard requirements. The findings provide optimized formulations and theoretical references for grouting reinforcement in karst tunnel projects.

## 1. Introduction

To meet the demands of urban underground space development, infrastructure construction in China’s karst regions faces severe geological challenges. Grouting technology plays a critical role in such projects, with grout performance optimization being a key focus for technological breakthroughs. Existing research primarily adopts two approaches to enhance performance: (1) chemical modification of traditional cement-based materials and (2) development and application of novel cementitious systems [1,2,3,4]. Li’s team developed new grout materials with superior engineering properties through the modification of conventional grouting materials [5] and the application of red mud-based geopolymer binders [6], with experimental and microstructural analyses confirming their technical advantages in specific engineering scenarios. Khayat [7] systematically investigated the effects of fly ash, slag, and other cementitious materials on the performance of cement-based grouts, finding that they significantly improved rheological properties (reduced plastic viscosity and yield stress), decreased bleeding, and enhanced compressive strength. Similarly, Mahmood et al. [8] demonstrated that polymer additives effectively regulate grout viscosity, yield stress, and other rheological parameters while enhancing compressive strength, providing solutions for engineering projects with special rheological requirements. Celik and Canakci [9] found that the incorporation of rice husk ash (RHA) not only optimized grout rheology but also improved compressive strength and reduced bleeding. Moreover, engineering practice has shown that polycarboxylate, naphthalene-based water reducers, and fly ash significantly enhance the performance of cement grouts [10]. Sonebi [11] further verified the strengthening effects of viscosity modifiers such as diutan gum and welan gum on fly ash-cement grouts, particularly in terms of stability, viscosity, and yield stress improvement.

Grout formulation research involves the interaction of multiple factors, and a well-designed experimental method can significantly enhance research efficiency and optimize mix proportions. Commonly used methods include orthogonal design [12,13,14], uniform design [15], neural network design [16], and full-factorial experiments [17], each with distinct characteristics in terms of experimental accuracy, sample size, and computational complexity, requiring appropriate selection based on research objectives. In multi-factor synergistic optimization studies, Hallal et al. [18] employed orthogonal experiments to investigate the combined effects of mineral admixtures and high-range water reducers. Through a limited combination of trials, they revealed the differentiated improvement in fluidity caused by varying material proportions, confirming that cement type and water-to-cement ratio are key factors in water reducer selection. Basu et al. [19] used response surface methodology to examine the effects of water–binder ratio and water reducer dosage on the properties of self-compacting concrete. Their mathematical model identified an optimal mix range that balanced fluidity and compressive strength. Liu et al. [20] analyzed the impact of nanoparticles on the fluidity, flexural, and compressive strength of cement mortar through experiments, combining SEM and XRD microstructural characterization. The results showed that appropriate nanoparticle content significantly enhanced compressive strength with minimal impact on fluidity. Zhao [21] conducted single-factor experiments to evaluate the effects of vibration mixing technology on the slump, compressive strength, and density of concrete with different mix proportions. The results indicated that this technique improved microstructure while maintaining high fluidity, thereby enhancing strength. In terms of engineering application optimization, Fang et al. [22] determined the optimal water-to-cement ratio and admixture dosage for semi-flexible pavement cement grouts through experimental design and SEM analysis, significantly improving fluidity and mechanical properties, with notable benefits for durability. Zhang et al. [23] optimized the combination of superplasticizers and retarders for sulfoaluminate cement (SAC) by conducting multi-index tests, including fluidity, setting time, and zeta potential. Their study improved fluidity while ensuring strength retention. Some researchers have also established grout performance prediction models using mathematical analysis and intelligent algorithms to guide engineering applications more efficiently. Chang and Zheng [24] developed a performance prediction model for ultra-high-performance concrete (UHPC) using regression analysis, identifying the influence coefficients of key parameters such as water–cement ratio. Svermova et al. [25] employed statistical models to quantify the improvement effects of limestone powder on grout rheological properties. Wu et al. [14] revealed the correlation between water–cement ratio and the performance of ultrafine cement grout through numerical simulations, while Du et al. [26] developed an intelligent prediction model that accurately forecasted the strength of cement backfill grout.

However, current research primarily focuses on the optimization of individual grout systems, lacking systematic comparisons of the applicability of different systems. To address this gap, this study investigates a karst cavern project in Xinjiang by designing five grout systems, optimizing the mix proportions through orthogonal experiments, and establishing a predictive model to fill the research void in grout system selection methods.

## 2. Grouting Material Test

### 2.1. Background of the Experiment

**Figure 1:**
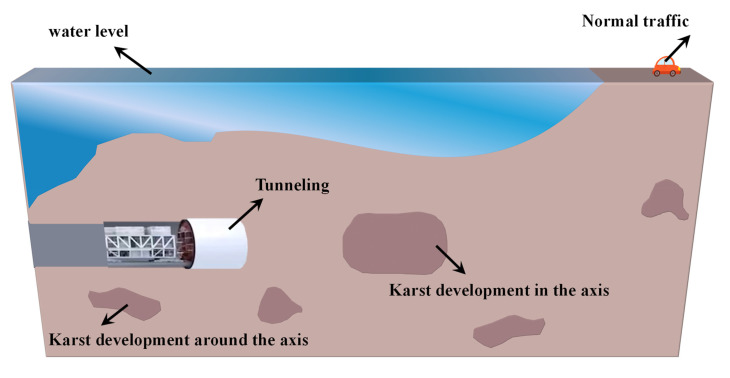
Longitudinal section of Xiaoshui Shield Tunnel Survey.

Karst landforms are widely distributed in Northwest China, and the developed karst caverns pose significant risks to tunnel construction. During preliminary surveys, if pronounced karst development is detected along the tunnel axis or in the surrounding areas (Figure 1), grouting reinforcement is required to ensure the safety of shield tunneling and the normal operation of surface transportation. Although traditional grouting techniques are widely applied, their effectiveness is often limited by grout formulation design. This study systematically analyzes the impact of different grout formulation proportions on grouting performance, aiming to optimize grouting systems for karst formations and provide theoretical references and technical guidance for engineering practice.

### 2.2. Experimental Design

The experimental design primarily focuses on evaluating the key performance indicators of the grout during the shield tunneling process, while also considering the material cost-effectiveness and construction operability. Based on material availability and cost advantages, clay and locally sourced lake mud were selected as the base materials. This choice also provides a reference for the resource utilization of shield tunnel spoil. To address potential mechanical performance limitations of pure clay-cement grout, a sand addition control group was specifically set up for comparison. The additives selected include sodium silicate and modified sodium silicate (HY-4, Hunan Hongyu Company, Changsha, China), which have been validated in engineering applications. As a control, the clay–cement–sand system adjusted its performance by varying the sand content without the addition of any additives. Based on pre-experiments and engineering experience, five grout systems are designed: (1) clay-cement-based grout (low cement content); (2) clay-cement-based grout (high cement content); (3) on-site wet clay-cement grout; (4) clay-cement with sand grout; and (5) lake mud-cement grout.

The L_9_(3^3^) orthogonal experimental design (Table 1) is used to optimize the test scheme, with specific parameter configurations shown in Table 2.

### 2.3. Test Materials and Test Methods

**Figure 2:**
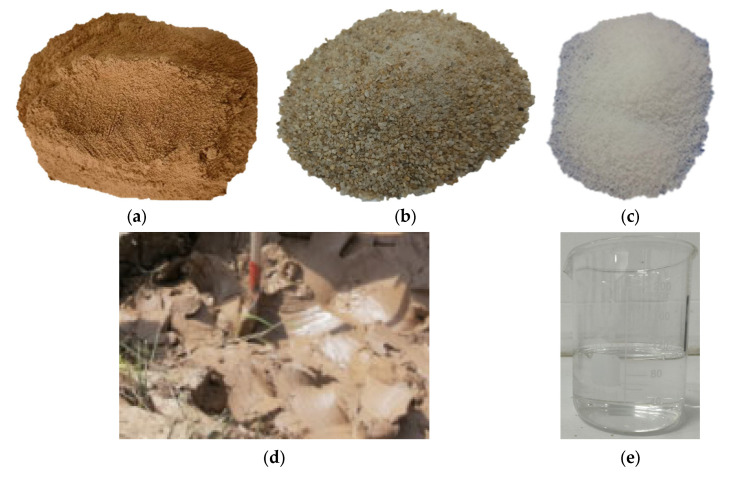
Test clay preparation procedure ((**a**): red clay sample, (**b**): standard sand samples, (**c**): HY-4 sample, (**d**): lake mud used for testing, (**e**): water glass samples).

**Figure 3:**
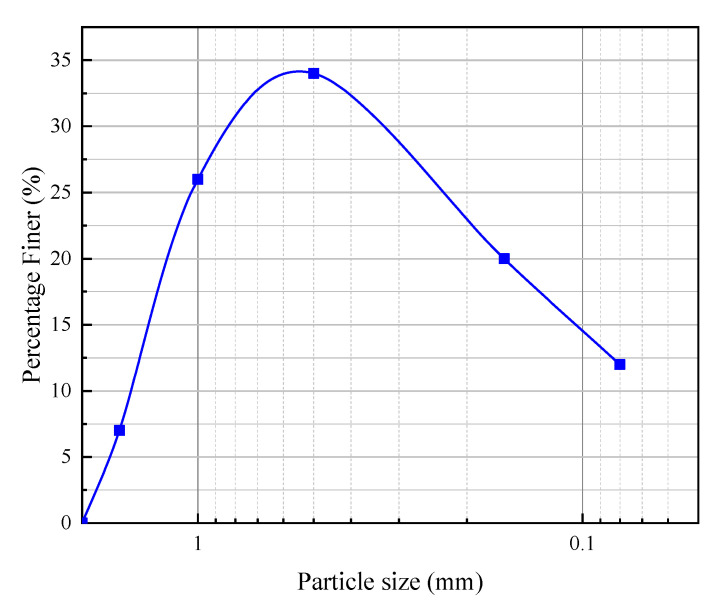
Standard sand grading curve.

In this experiment, soil samples were prepared using the drying and sieving method. After the grout was prepared, samples were molded using a standard mold with dimensions of 70.7 mm × 70.7 mm × 70.7 mm. To analyze the effects of different materials and dosages on the grout system’s performance parameters, the experiment selected clay, sand, lake mud, and additives (sodium silicate and HY-4). The clay was sourced from the typical red clay in the Yuelu Mountain area (Figure 2). The specific gravity of the lake mud is 2.671, with a compression coefficient of 0.893 MPa^−1^, a liquid limit of 41.18%, and a plastic limit of 22.08%, as determined by laboratory tests. The aggregate used was ISO standard sand produced by Xiamen Aiso Standard Sand Co., Ltd. (Xiamen, China), with the particle grading curve shown in Figure 3.

**Figure 4:**
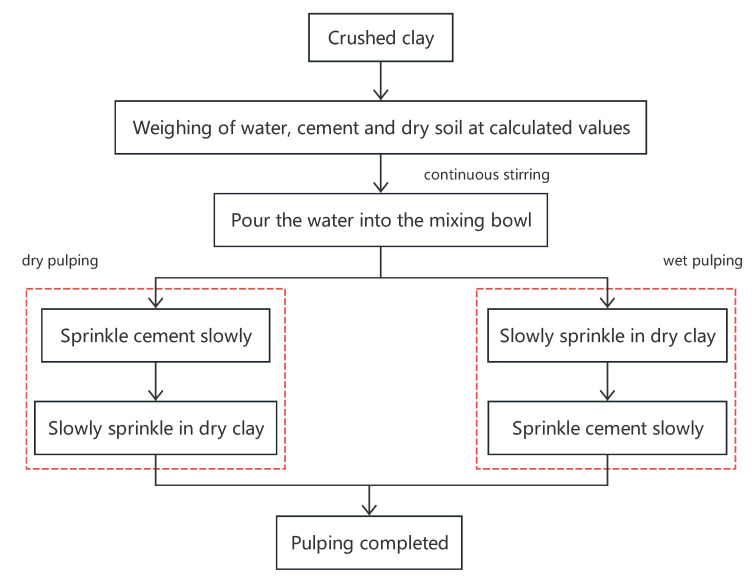
Preparation process of paste.

The formulation process was divided into dry and wet methods, with specific steps outlined in Figure 4.

## 3. Analysis of Experimental Results

### 3.1. Time-Dependent Strength of Grout Slurry

**Figure 5:**
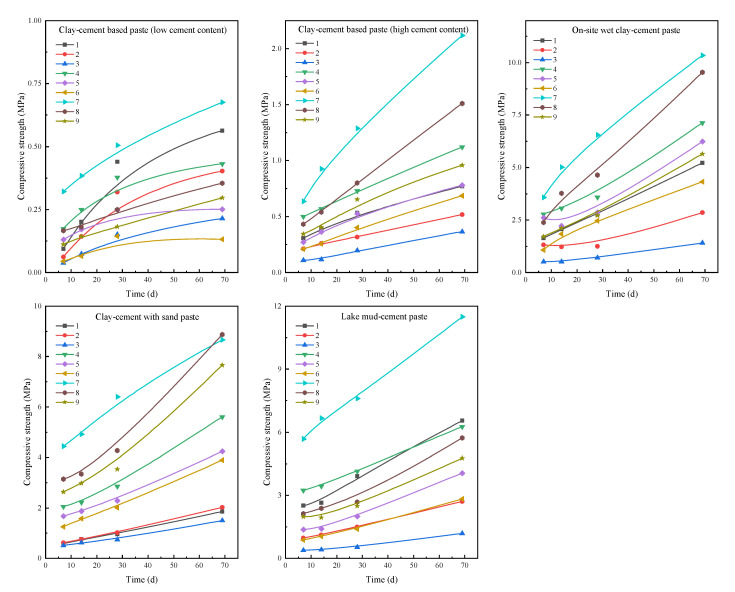
Time-dependent compressive strength curve of grout concretions.

The compressive strength of the grout-solidified body is a key indicator for evaluating the grouting reinforcement effectiveness, particularly in enhancing the reinforcement and impermeability of the grouting curtain. Figure 5 shows the variation in compressive strength of the grout-solidified bodies over time for each system. For ease of description, the cement content is denoted as factor A, water content as factor B, and the dosage of additives/sand as factor C.

The overall trend of compressive strength for the grout solidified bodies is as follows: (e) > (c) > (d) > (b) > (a). The lake mud-cement grout-solidified body exhibited the highest average compressive strength within the testing range, with the clay-cement with sand grout showing similar strength, while the on-site wet clay-cement grout had slightly lower compressive strength compared to the first two. The compressive strength of clay-cement-based grout (low cement content) was significantly lower than that of the other systems, but its preparation process is simple and does not require large amounts of solvents or liquid treatment, making it suitable for simple grouting projects.

When each grout system is analyzed individually, experiment No. 7 (A3B1C3 formulation) shows a significantly higher compressive strength than other formulations, indicating that an increase in cement content significantly enhances the compressive strength of the solidified body, while lower water content also effectively improves strength. The use of sodium silicate additives and the incorporation of standard sand both significantly enhanced the compressive performance of the solidified body. Although the A3B1C3 formulation performs excellently in compressive strength, whether it is the optimal combination for the system still requires further validation.

### 3.2. Range Analysis in Orthogonal Design

**Figure 6:**
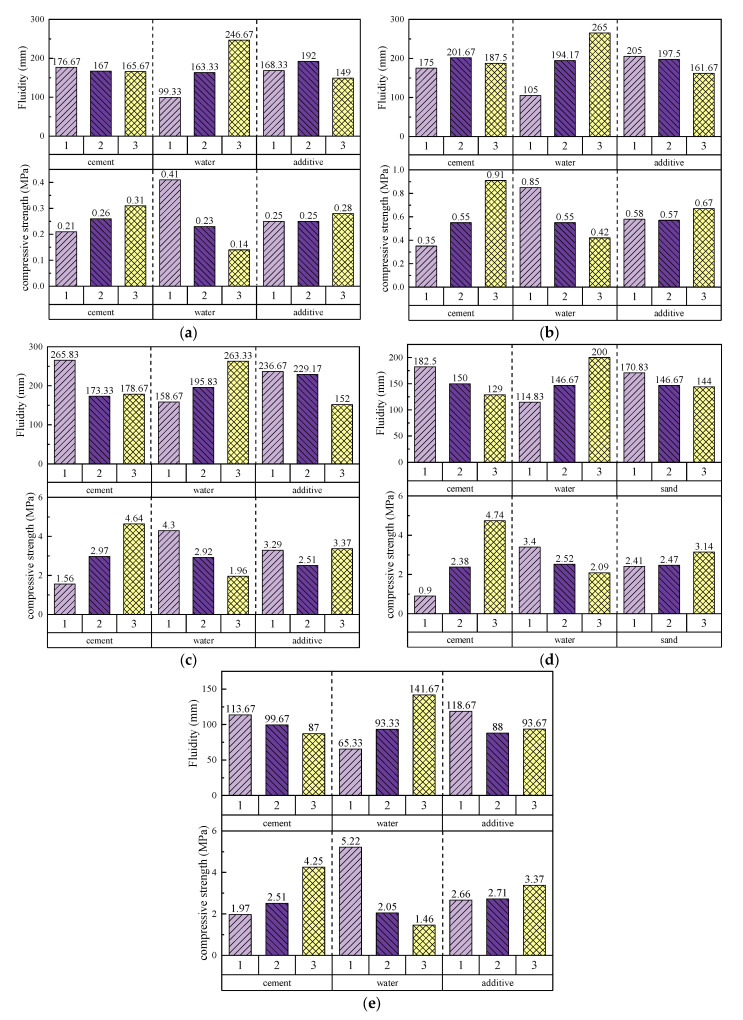
Water averages for each of the influencing factors for the five pastes ((**a**): clay-cement-based paste (low cement content); (**b**): clay-cement-based paste (high cement content); (**c**): on-site wet clay-cement paste; (**d**): clay-cement with sand paste; (**e**): lake mud-cement paste).

To evaluate the results of the orthogonal experiment, analysis of variance (ANOVA) was performed on the 28-day compressive strength data of all the solidified grouts. The range values (R-values) for each factor level were calculated through range analysis (Table 3) to quantify the impact of each factor on flowability and compressive strength. The results show that water content is the dominant factor affecting the flowability of all grout systems, although its importance varies with the system composition. The following is a detailed analysis of each grout system:

In the clay-cement-based grout (low cement content) system (Figure 6a), the best flowability was achieved with the A1B3C2 combination (low cement, high water content, medium additives), indicating that high water content significantly improves flowability. The range analysis shows that water content has the most significant impact on flowability, followed by additives, while cement content has the least influence. In terms of compressive strength, the A3B1C3 combination (high cement, low water content, sodium silicate) performed the best. Sodium silicate performed exceptionally well in enhancing strength but significantly reduced flowability. The HY-4 additive, although improving flowability, had no significant effect on strength.

In the clay-cement-based grout (high cement content) system (Figure 6b), maximum flowability was observed with the A2B3C1 combination (medium cement, high water content, low additives). Unlike the low cement system, increasing cement content initially improved flowability, but at higher levels, the interaction between cement particles led to reduced flowability. For compressive strength, the A1B1C3 combination (lowest cement, lowest water content, sodium silicate) performed the best. Sodium silicate significantly increased strength but reduced flowability. Overall, cement content showed a positive correlation with strength, while water content showed a negative correlation with strength, reflecting the trade-off between flowability and mechanical properties.

In the on-site wet clay-cement grout system (Figure 6c), due to the higher moisture content of the raw materials, the best flowability was achieved with the A1B3C1 combination (low cement, high water content, no additives). The range analysis indicated that the influences of cement, water, and additives on flowability were comparable. In terms of compressive strength, the A3B1C3 combination (high cement, low water content, sodium silicate) showed the best performance. Notably, the HY-4 additive, although increasing grout density, reduced the efficiency of hydration reactions, leading to a decrease in compressive strength. Therefore, in this system, HY-4 is more suitable for impermeability applications rather than structural reinforcement.

In the clay-cement with sand grout system (Figure 6d), standard sand was introduced to replace part of the additives to enhance mechanical properties. The best flowability was achieved with the A1B3C1 combination (low cement, high water content), while the compressive strength peaked with the A3B1C3 combination (high cement, low water content). The addition of sand improved strength within an appropriate range, but excessive sand reduced flowability. This is because sand particles form a skeletal structure in the grout, enhancing compressive strength while reducing injectability. This suggests that in engineering applications, a balance must be struck between the formation of a solid skeleton and injectability to ensure reinforcement effectiveness and construction feasibility.

In the lake mud-cement grout system (Figure 6e), the best flowability was achieved with the A1B3C1 combination (low cement, high water content), while the compressive strength peaked with the A3B1C3 combination (high cement, low water content). Flowability was positively correlated with water content, while compressive strength was inversely related to water content. Higher water content led to more pores in the solidified grout, significantly reducing strength, while higher cement content enhanced the bonding between particles, improving strength. This indicates that in the lake mud-cement grout system, the cement content plays a dominant role in strength development.

### 3.3. Analysis of 3D Scatter Plots

**Figure 7:**
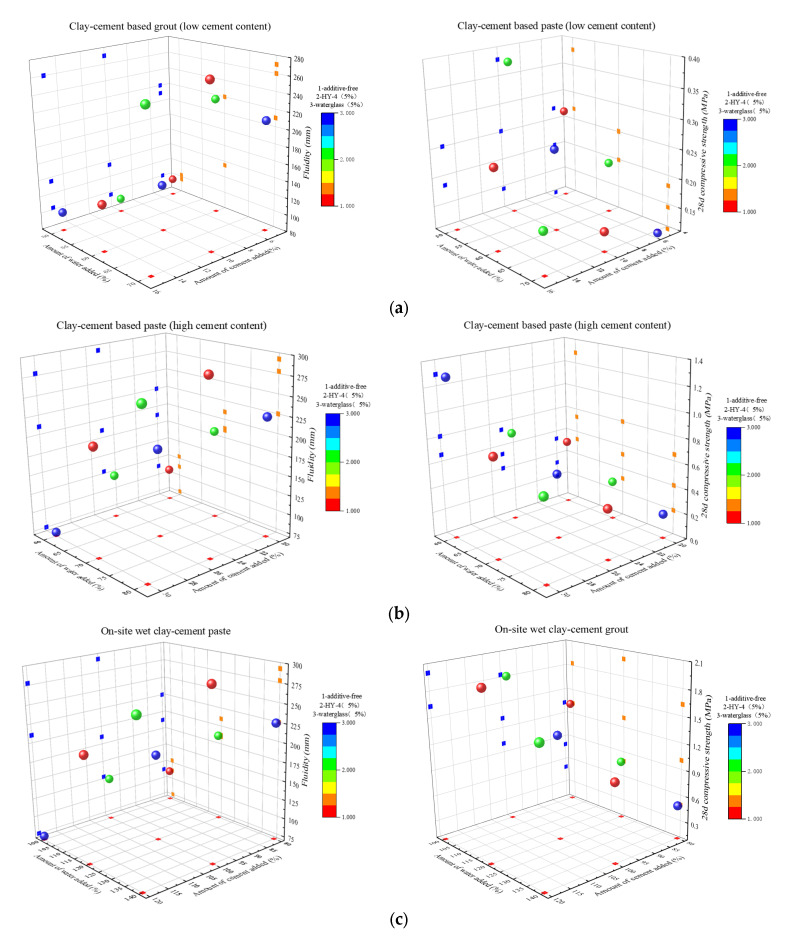

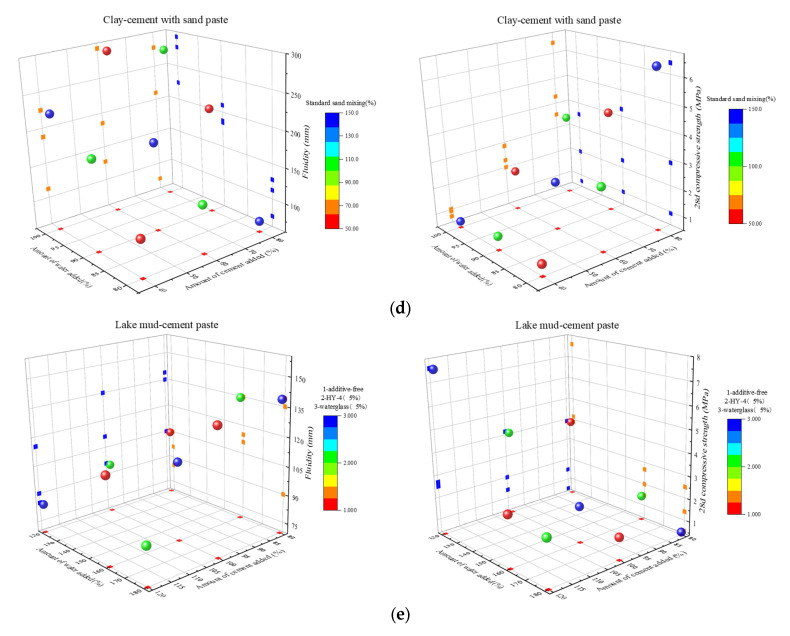
Three-dimensional scatter plot of paste for each system ((**a**): clay-cement-based paste (low cement content); (**b**): clay-cement-based paste (high cement content); (**c**): on-site wet clay-cement paste; (**d**): clay-cement with sand paste; (**e**): lake mud-cement paste).

Three-dimensional scatter plots were used to analyze the effects of various factors on the grout properties (Figure 7), where the x and y axes represent water content and cement content, and the third variable is represented by a color gradient.

Figure 7a shows that the incorporation of HY-4 significantly improved the flowability of the grout. When the flowability was >200 mm, the systems with 5% HY-4 had the highest proportion (two data points), mostly distributed in the high cement content region, indicating that the water-to-cement ratio is the key parameter controlling the flowability of grouts with low cement content (5–15%). The optimal compressive strength was observed under the conditions of 5% HY-4 and the lowest water content. The data distribution showed the pattern “high in the west, low in the east, high in the north, low in the south”, indicating that reducing water content, increasing cement content, and adding HY-4 contribute to achieving the best strength.

The data distribution pattern in Figure 7b is similar to that in Figure 7a, with flowability mainly concentrated around 200 mm. However, the data points in the middle of the graph are noticeably higher, and the number of data points below 120 mm decreased. The flowability of the grout with 5% sodium silicate remained stable at around 200 mm, confirming the significant role of additives in improving flowability in high cement systems. Strength analysis shows that the maximum compressive strength appeared in the region with low water content, high cement content, and 5% sodium silicate, showing a significant negative correlation between water content and strength. Notably, sodium silicate had a much stronger enhancement effect compared to HY-4, which even showed a strength suppression effect in high cement content systems.

Figure 7c shows that, without additives, the grout had the highest flowability, followed by the system with 5% HY-4, while the system with 5% sodium silicate had the lowest flowability. This indicates that additives (especially sodium silicate) reduce the flowability of the system. Strength analysis shows that the 5% sodium silicate system, with the lowest flowability, had the highest strength, followed by the 5% HY-4 system, and the system without additives had the lowest strength. In addition, increasing cement content significantly enhanced strength, and this effect was more pronounced in the on-site wet clay-cement grout system than in the traditional system.

In the clay-cement with sand grout system (Figure 7d), the sand content significantly impacted both flowability and compressive strength. When the sand content increased to 150%, the flowability of the grout was significantly reduced compared to the 50% and 100% ratios, and combinations with low water content, low cement, and low sand content deteriorated the grout performance. Compressive strength analysis showed that the maximum strength occurred in the combination with the highest cement content, the lowest water content, and 150% sand content. Notably, the effects of cement content and water content on strength showed an interaction: with high cement content, increasing water content significantly reduced strength, whereas with low cement content, changes in water content had a relatively small effect on strength. Based on this, it is recommended in engineering applications to use high cement-to-water ratios for high strength and to reduce cement content and increase water content appropriately to optimize comprehensive performance when strength requirements are lower.

Figure 7e shows that the flowability of lake mud-cement grout is significantly lower than that of clay-based systems, likely due to the network structure formed by the organic matter in the lake mud, which enhances particle cohesion and reduces free water. The impact of additives is ratio-dependent: at high cement content, both 5% HY-4 and 5% sodium silicate reduce flowability, while at low cement content, they improve flowability. The highest compressive strength in this system reached 7.5 MPa, outperforming some clay-cement with sand grout systems, with 5% sodium silicate showing the most significant enhancement effect under conditions of high cement and low water content, demonstrating good potential for engineering applications.

Analysis indicates that for clay-cement-based grout, water-to-cement ratio dominates flowability at low cement content, and HY-4 can significantly improve flowability; at high cement content, sodium silicate has a significant effect on both flowability and strength, and the maximum strength can be achieved with low water content. In the on-site wet clay-cement grout system, the flowability is best without additives, but additives can improve strength. In the clay-cement with sand grout system, sand content significantly affects performance, and an optimal addition amount exists. In the lake mud-cement grout system, although flowability is lower, the compressive strength can reach up to 7.5 MPa, outperforming some clay-cement with sand grout systems, particularly with the significant enhancement effect of sodium silicate additives.

## 4. Grout Performance Prediction Model

### 4.1. Model Construction

This section employs a multiple linear regression model to quantitatively assess the impact of various variables on the flowability and compressive strength of the grout and to construct the corresponding prediction model. In the model, water content and cement content are treated as continuous variables, while two dummy variables, D1 and D2, are introduced to represent the categorical variables of the three types of additives. The dummy variables take values of 0 or 1 to indicate the different combinations of additives. Table 4 lists the correspondence between the dummy variable values and the additive combinations.

The final regression model can be expressed as Equation (1):

(1) $$Y(w/f_{c,28})=\beta_{0}+\beta_{1}X_{1}+\beta_{2}X_{2}+\beta_{3}D_{1}+\beta_{4}D_{2}$$

In the equation, *Y* represents the dependent variable (flowability or 28-day compressive strength); *β*_0_ is the intercept term; *β*_1_ and *β*_2_ are the regression coefficients for water content and additive dosage, respectively, indicating the change in *Y* for each unit increase of the corresponding variable; and *β*_3_ and *β*_4_ are the regression coefficients for the dummy variables (*D*_1_ and *D*_2_), representing the differences relative to the reference group (*D*_0_).

### 4.2. Model Fitting Equations

Based on the experimental data, regression analysis was conducted using SAS 9.4 EAS statistical software to establish linear regression equations for the five grout systems. The flowability and compressive strength prediction models for the five grout systems are shown in Table 5.

### 4.3. Regression Model Evaluation and Validation

To ensure the accuracy and reliability of the model, two methods are used for systematic evaluation: statistical validation and independent experimental validation.

#### 4.3.1. Statistical Validation

First, the statistical validity of the model was verified through regression diagnostics and hypothesis testing. The multicollinearity test results (Table 6) show that the variance inflation factors (VIFs) for all independent variables are below 10 (ranging from 1.00 to 1.33), and the tolerance values are greater than 0.1 (ranging from 0.75 to 1.00), indicating that there is no severe multicollinearity and that the regression coefficients are stable. The Durbin–Watson statistic ranges from 1.3 to 3.1, with most values close to 2, indicating no significant autocorrelation in the residuals, thus satisfying the assumptions of linear regression. In terms of model fit, the lowest coefficient of determination (R^2^) is 0.74 (for the compressive strength model of the clay-cement with sand grout system), while the R^2^ values for the other models range from 0.80 to 0.95, suggesting that the models can explain 74% to 95% of the variation in the dependent variables and provide good fits. Additionally, the analysis of variance (ANOVA) results show that the *p*-values for all models are less than 0.05, indicating that the regression coefficients are statistically significant.

#### 4.3.2. Independent Single-Factor Experimental Validation

**Figure 8:**
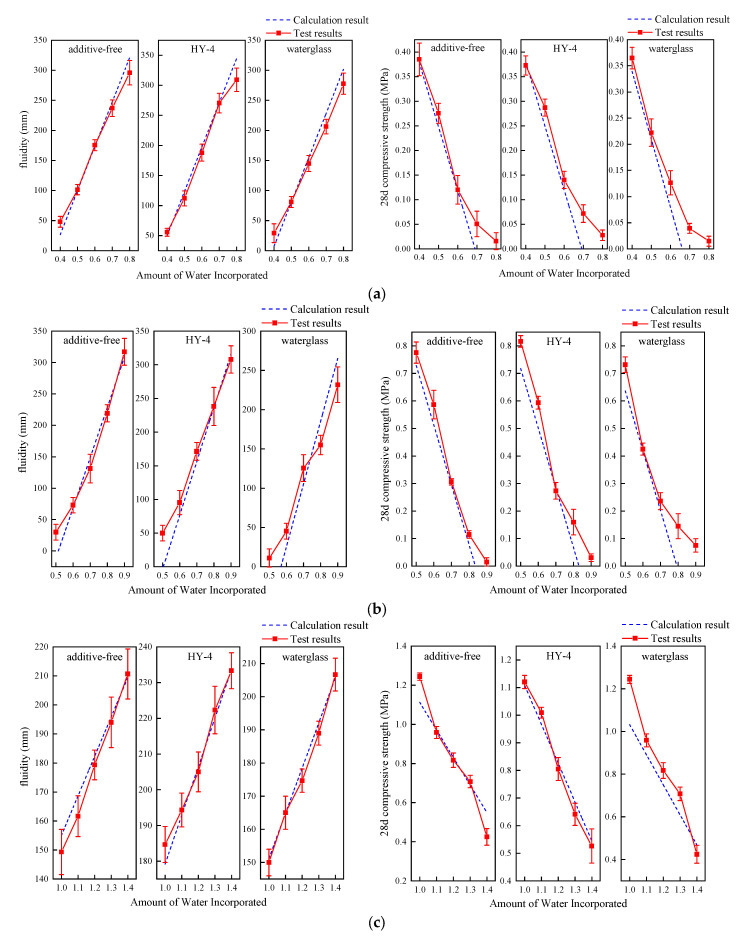

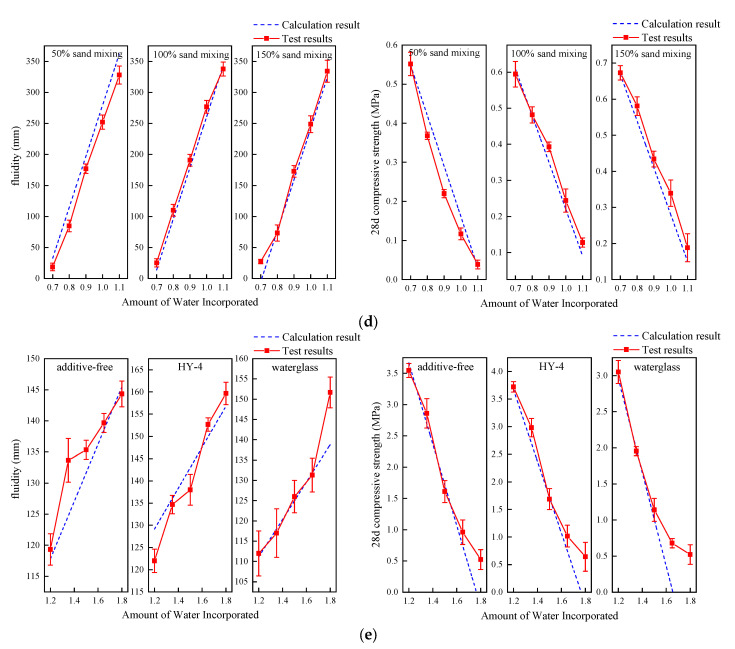
Comparison of calculated and experimental values of the paste prediction model ((**a**): clay-cement-based paste (low cement content); (**b**): clay-cement-based paste (high cement content); (**c**): on-site wet clay-cement paste; (**d**): clay-cement with sand paste; (**e**): lake mud-cement paste).

To further validate the predictive performance of the model, a single-factor control experiment was designed, and the experimental measurements were compared with the model predictions (Figure 8). The results show that the models for the five grout systems have a high degree of fit, with the overall trends being consistent with the experimental data, providing strong reference value.

In the clay-cement-based grout (low cement content) system, the model predicts flowability quite accurately, especially at low water content, where the fit is significant. However, when the water amount increases to 0.8, the model’s prediction is slightly lower than the actual measurement, although the overall trend remains consistent. Regarding compressive strength, the model’s prediction shows some deviation at high water content, with experimental results indicating that some samples still retain a certain strength, suggesting limitations in the model within this range.

The clay-cement-based grout (high cement content) system exhibits a similar pattern. When the water content is less than 0.7, the model’s predictions are usually lower than the actual values, and vice versa. This discrepancy is particularly evident when 5% sodium silicate is added, where the linear relationship is not clear.

In the on-site wet clay-cement grout system, the flowability trend predicted by the model is generally consistent with the experimental results, though there is greater fluctuation in the measured values, which may be attributed to the instability of the on-site preparation process. The compressive strength prediction is relatively accurate when the water content is between 1.1 and 1.3, but for extreme water content (either too low or too high), the model’s prediction is either too low or too high.

In the clay-cement with sand grout system, both the flowability and compressive strength predictions are highly consistent with the experimental measurements, indicating that the model effectively reflects the impact of sand content on the system’s performance.

In the lake mud-cement grout system, although there is some deviation between the model and the measured values, the overall trend is consistent. The prediction is most accurate when 5% sodium silicate is added. However, slight deviations are observed in the samples with no additives and those with 5% HY-4. It is noteworthy that at high water content, the model’s prediction for compressive strength is lower than the actual measurement, which warrants further attention.

## 5. Discussion and Interpretation

According to the “Highway Karst Tunnel Design and Construction Technical Code” (JTG/T 3373-2024) and the “Shield Tunnel Engineering Design Standard” (GB/T 51438-2021) [27,28], cement-based grout is recommended for karst areas, and the unconfined compressive strength of the reinforced strata should not be less than 1 MPa. The results of this study show that the lake mud-cement grout (A3B1C3 formulation) achieved a 28-day compressive strength of 4.74 MPa, and the on-site wet clay-cement grout (A2B3C1 formulation) achieved a strength of 1.1 MPa, both of which meet and exceed the standard requirements, verifying their applicability in karst reinforcement. The incorporation of clay and lake mud not only ensures engineering performance (≥1 MPa) but also reflects the environmental and economic advantages of using locally sourced materials.

In terms of international standards, ASTM C939 [29] specifies that the fluidity of grouting materials should be between 150 and 300 mm. In this study, the fluidity of the on-site wet clay-cement grout (A2B3C1 formulation) reached 292.5 mm, fully meeting the grouting requirements for high permeability strata. The lake mud-cement grout (A3B1C3 formulation) had a fluidity of 152 mm, which is at the lower limit of the standard, but its higher compressive strength (4.74 MPa) makes it more suitable for scenarios with low groundwater flow rates and stability prioritization. In addition, the European concrete standard EN 206 [30] emphasizes the “performance-oriented” mix design principle. The optimized model constructed through orthogonal testing and linear regression analysis in this study aligns well with this principle, providing theoretical support for scientific decision-making in slurry formulation. Based on the results of the orthogonal experiments, the recommended formulations for each slurry system and their applicable engineering scenarios are summarized in Table 7.

Despite the progress made in this study, there are still several limitations. First, the variable ranges in the orthogonal experiment were primarily based on empirical values, which may limit the accuracy and reliability of the experimental results. Future research could use optimization tools such as Response Surface Methodology (RSM) to improve the experimental design and enhance the precision and credibility of the results. Second, as this study focused on multiple grout systems, the number of experimental groups for each system was relatively limited. Future studies could increase the number of experimental groups for individual systems to more comprehensively evaluate their performance in different engineering scenarios. Third, this study did not include microstructure analysis, which is a significant limitation. Subsequent research could incorporate techniques such as scanning electron microscopy (SEM) to characterize the microstructural features of grout, thereby revealing its performance mechanisms. Furthermore, future research could combine computer vision technologies to enhance the automated analysis of grout flow patterns, pore distribution, and uniformity [31,32]. For example, deep learning-based semantic segmentation models (such as DeepLab) could be used to accurately identify grout diffusion paths, while efficient convolutional neural network models (such as EfficientNet) could automatically extract and classify features related to pore distribution and grout uniformity. These technologies could enable real-time, automated detection and quantitative analysis, providing new methods for optimizing grout systems and improving their performance in engineering applications.

## 6. Conclusions

During shield tunnel construction, significant karst development was encountered along the tunnel axis and in the surrounding area. To address this challenge, this study proposes multiple grouting schemes to overcome the limitations of existing research regarding grout system selection. Five different grout formulations were designed and systematically analyzed through orthogonal experiments to evaluate their key performance characteristics and identify the optimal formulations for engineering applications. Based on this, a predictive model was established using linear regression, and its accuracy was validated through independent single-factor experiments. This study yields the following conclusions:

(1) Based on the experimental test results of the five grout systems, the lake mud-cement grout exhibited the highest average compressive strength within the test range, followed by the clay-cement grout with sand, while the on-site wet clay-cement grout was slightly lower than the former two. The compressive strength of the dry-prepared clay-cement grout was significantly low, but the preparation process was simple and suitable for simple grouting projects. Range analysis indicated that the water-to-cement ratio was the dominant factor affecting flowability, and its importance varied with the system composition. The appropriate addition of sand helps to improve strength, but excessive amounts significantly reduce flowability. The effect of additives showed a certain degree of system dependence. HY-4 effectively improved flowability, especially for low-cement systems, but its strength enhancement was limited. Sodium silicate could significantly increase strength but may reduce flowability, making it suitable for high-cement and low-water conditions. Grout performance is affected by the interaction of sand content, cement content, and water-to-cement ratio, and the formulation needs to be optimized considering the characteristics of additives to meet engineering requirements.
(2) Based on experimental data, mathematical modeling was employed for analysis, and its accuracy and reliability were evaluated through statistical validation and independent experimental validation. The results show that the constructed model has a good fit, with the coefficient of determination (R^2^) ranging from 0.74 to 0.95, explaining 74% to 95% of the variation in the dependent variable. The regression coefficients are statistically significant (*p* < 0.05), there is no severe multicollinearity, and the residuals show no significant autocorrelation, thus satisfying the assumptions of linear regression. Independent single-factor experimental validation further proves that the model has strong predictive capability. The model fitting for the five grout systems is high, and the overall trends are consistent with the experimental data, providing valuable reference. The model performs more accurately in the low water-to-cement ratio range, but there is a slight deviation at high water-to-cement ratios, with the predicted compressive strength for some systems being slightly lower than the measured values. The consistency of predictions for clay-cement with sand grout and lake mud-cement grout is relatively high, especially at a 5% sodium silicate addition, where the model performs the best. Overall, the model effectively reflects the variations in flowability and compressive strength of different grout systems.
(3) Based on the research results, optimized grout formulations and their applicable engineering scenarios are recommended. The lake mud-cement grout (A3B1C3 formulation) has a 28-day compressive strength of 4.74 MPa, making it suitable for reinforcement projects where low groundwater flow and stability are prioritized. The on-site wet clay-cement grout (A2B3C1 formulation) has a strength of 1.1 MPa and flowability of 292.5 mm, making it suitable for high permeability strata. Both formulations exceed the typical standard requirements, validating their applicability in grouting reinforcement in karst areas. The limitations of this study include the limited number of experimental groups, and future work may further optimize the design by incorporating computer vision and deep learning techniques.

### Research Prospects

The optimization of paste systems and their micro-mechanisms is a complex research field, and this paper comprehensively analyzes the effects of different paste system ratios on their fluidity and compressive strength. In the future, in-depth research can be carried out in the following aspects:

Firstly, the range of values in the orthogonal test is too dependent on empirical values, which limits the accuracy of the experimental results to a certain extent. In the future, optimization tools such as Response Surface Methodology can be combined to further optimize the experimental range of values, and the optimal values of key variables can be selected through a more systematic experimental design method. This can not only reduce the experimental workload but also improve the accuracy of the experimental results, making the optimization of the paste ratio more scientific and reasonable.

Secondly, since this paper focuses on the reasonable ratio interval of multiple paste systems, the number of test groups for single-system paste is slightly insufficient. Future research can increase the number of test groups for single-system pastes to explore the specific performance of each paste system, especially its applicability in different engineering application scenarios. At the same time, the optimization of paste ratios under different water–cement ratios can also be investigated to further improve the fluidity and compressive strength of the pastes.

Finally, micro-mechanistic studies are crucial to understanding paste performance. In this paper, there is a lack of in-depth analysis of the microstructure of the paste system. In the future, the internal structure of the paste system can be characterized using scanning electron microscopy (SEM), among other methods, to explore the microstructure of paste solidification and its formation mechanism in depth under different ratios, providing theoretical support for the further optimization of paste ratios.

## Figures and Tables

**Table 1:** Table of orthogonal experimental designs.

No.	Admixture/%		
	Cement	Water	Additives/Sand
1	1	1	1
2	1	2	2
3	1	3	3
4	2	1	2
5	2	2	3
6	2	3	1
7	3	1	3
8	3	2	1
9	3	3	2

**Table 2:** Grout composition mixing table. (a) Clay-cement-based grout (low cement content). (b) Clay-cement-based grout (high cement content). (c) On-site wet clay-cement grout. (d) Clay-cement with sand grout. (e) Lake mud-cement grout.

(a)			
Factor	Level		
	1	2	3
Clay/%	100	100	100
A: Cement/%	20	25	30
B: Water/%	60	70	80
C: Additives/%	0	HY-4 (5%)	waterglass (5%)
**(b)**			
**Factor**	**Level**		
	**1**	**2**	**3**
Clay /%	100	100	100
A: Cement /%	5	10	15
B: Water /%	50	60	70
C: Additives /%	0	HY-4 (5%)	waterglass (5%)
**(c)**			
**Factor**	**Level**		
	**1**	**2**	**3**
Clay/%	100	100	100
A: Cement/%	80	100	120
B: Water/%	100	120	140
C: Additives/%	0	HY-4 (5%)	waterglass (5%)
**(d)**			
**Factor**	**Level**		
	**1**	**2**	**3**
Clay /%	100	100	100
A: Cement /%	40	60	80
B: Water /%	80	90	100
C: Sand /%	50	100	150
**(e)**			
**Factor**	**Level**		
	**1**	**2**	**3**
lake mud /%	100	100	100
A: Cement /%	80	100	120
B: Water /%	120	150	180
C: Additives /%	0	HY-4 (5%)	waterglass (5%)

**Table 3:** Statistical table of R-value for each factor.

R-Value Statistics	Fluidity			Compressive Strength		
	Cement	Water	Additives/Sand	Cement	Water	Additives/Sand
Clay-cement-based paste (low cement content)	11	147.33	43	0.1	0.27	0.04
Clay-cement-based paste (high cement content)	26.67	160	43.33	0.57	0.43	0.1
On-site wet clay-cement paste	92.5	104.67	84.67	0.1	0.11	0.02
Clay-cement with sand paste	53.5	85.17	26.83	3.84	1.31	0.73
Lake mud-cement paste	26.67	76.33	30.67	2.28	3.75	0.71

**Table 4:** Correspondence between dummy variables and admixture type.

No.	D_1_	D_2_	Additive
1	0	0	additive-free
2	1	0	HY-4 (5%)
3	1	1	waterglass (5%)

**Table 5:** Regression models for the performance of the five grout systems.

Paste System	Implicit Variable	Regression Fitted Formula
Clay-cement-based paste (low cement content)	fluidity	*Y_a_*(*w*) = −262.667 − 110*C* + 736.7*W* + 23.667*A_HY_*_-4_ − 19.333*A_SS_*
	compressive strength	*Y_a_*(*f_c_*_,28_) = 0.948 + 1.012*C* − 1.3*W* + 0.002*A_HY_*_-4_ − 0.038*A_ss_*
Clay-cement-based paste (high cement content)	fluidity	*Y_b_*(*w*) = −386.25 − 125C + 800*W* + 7.5*A_HY_*_-4_ − 43.333*A_ss_*
	compressive strength	*Y_b_*(*f_c_*_,28_) = 0.683 + 5.653C − 2.17*W* − 0.011*A_HY_*_-4_ − 0.092*A_SS_*
On-site wet clay-cement paste	fluidity	*Y_c_*(*w*) = 64.417 − 57.083C + 136.25*W* + 24.167*A_HY_*_-4_ − 3.833*A_ss_*
	compressive strength	*Y_c_*(*f_c_*_,28_) = 2.087 + 0.54*C* − 1.41*W* − 0.004*A_HY_*_-4_ − 0.08*A_SS_*
Clay-cement with sand paste	fluidity	*Y_d_*(*w*) = −495.808 − 70.444*C* + 822.554*W* − 38.172*S*
	compressive strength	*Y_d_*(*f_c_*_,28_) = 1.302 + 0.234*C* − 1.295*W* + 0.12*S*
Lake mud-cement paste	fluidity	*Y_e_*(*w*) = 121.857 − 74.2C + 46.1*W* + 11.333*A_HY_*_-4_ − 6.667*A_ss_*
	compressive strength	*Y_e_*(*f_c_*_,28_) = 6.908 + 5.7*C* − 6.5*W* + 0.048*A_HY_*_-4_ − 0.71*A_ss_*

**Table 6:** Regression diagnostic statistics for paste systems.

Paste System	Implicit Variable	R^2^	P	VIF	Durbin-Watson
Clay-cement-based paste (low cement content)	fluidity	0.896	0.030	1/1/1.333/1.333	2.037
	compressive strength	0.953	0.007	1/1/1.333/1.333	2.678
Clay-cement-based paste (high cement content)	fluidity	0.975	0.002	1/1/1.333/1.333	1.378
	compressive strength	0.945	0.009	1/1/1.333/1.333	2.065
On-site wet clay-cement paste	fluidity	0.792	0.005	1/1/1.333/1.333	2.055
	compressive strength	0.953	0.007	1/1/1.333/1.333	2.678
Clay-cement with sand paste	fluidity	0.869	0.012	1.261/1.261/1.523	3.178
	compressive strength	0.935	0.002	1.261/1.261/1.523	3.016
Lake mud-cement paste	fluidity	0.74	0.049	1/1/1.333/1.333	2.533
	compressive strength	0.909	0.024	1/1/1.333/1.333	2.462

**Table 7:** Recommended grout proportions and applications.

	Liquidity-Driven	Strength-Driven
Clay-cement-based paste (low cement content)	A1B3C2	A3B1C3
Clay-cement-based paste (high cement content)	A2B3C1	A3B1C3
On-site wet clay-cement paste	A1B3C1	A3B1C3
Clay-cement with sand paste	A1B3C1	A3B1C3
Lake mud-cement paste	A1B3C1	A3B1C3
Suggested Engineering Scenarios	Uniformly distributed and penetration demanding works	Works where stability and settlement characteristics are critical

## Data Availability

The original contributions presented in this study are included in the article. Further inquiries can be directed to the corresponding author.
